# Evidence-based practice educational intervention studies: a systematic review of what is taught and how it is measured

**DOI:** 10.1186/s12909-018-1284-1

**Published:** 2018-08-01

**Authors:** Loai Albarqouni, Tammy Hoffmann, Paul Glasziou

**Affiliations:** Centre for Research in Evidence Based Practice (CREBP), Faculty of Health Science and Medicine, Bond University, Gold Coast, Australia

**Keywords:** Evidence-based practice, Assessment tools, Teaching curriculum

## Abstract

**Background:**

Despite the established interest in evidence-based practice (EBP) as a core competence for clinicians, evidence for how best to teach and evaluate EBP remains weak. We sought to systematically assess coverage of the five EBP steps, review the outcome domains measured, and assess the properties of the instruments used in studies evaluating EBP educational interventions.

**Methods:**

We conducted a systematic review of controlled studies (i.e. studies with a separate control group) which had investigated the effect of EBP educational interventions. We used citation analysis technique and tracked the forward and backward citations of the index articles (i.e. the systematic reviews and primary studies included in an overview of the effect of EBP teaching) using Web of Science until May 2017. We extracted information on intervention content (grouped into the five EBP steps), and the outcome domains assessed. We also searched the literature for published reliability and validity data of the EBP instruments used.

**Results:**

Of 1831 records identified, 302 full-text articles were screened, and 85 included. Of these, 46 (54%) studies were randomised trials, 51 (60%) included postgraduate level participants, and 63 (75%) taught medical professionals. EBP Step 3 (critical appraisal) was the most frequently taught step (63 studies; 74%). Only 10 (12%) of the studies taught content which addressed all five EBP steps. Of the 85 studies, 52 (61%) evaluated EBP skills, 39 (46%) knowledge, 35 (41%) attitudes, 19 (22%) behaviours, 15 (18%) self-efficacy, and 7 (8%) measured reactions to EBP teaching delivery. Of the 24 instruments used in the included studies, 6 were high-quality (achieved ≥3 types of established validity evidence) and these were used in 14 (29%) of the 52 studies that measured EBP skills; 14 (41%) of the 39 studies that measured EBP knowledge; and 8 (26%) of the 35 studies that measured EBP attitude.

**Conclusions:**

Most EBP educational interventions which have been evaluated in controlled studies focus on teaching only some of the EBP steps (predominantly critically appraisal of evidence) and did not use high-quality instruments to measure outcomes. Educational packages and instruments which address all EBP steps are needed to improve EBP teaching.

**Electronic supplementary material:**

The online version of this article (10.1186/s12909-018-1284-1) contains supplementary material, which is available to authorized users.

## Background

Evidence-Based Practice (EBP) is the integration of the best available research evidence with clinical expertise and patient’s unique values and preferences (i.e. personal concerns, expectations, cultural influences and individual characteristics during the clinical encounter) [1]. The Institute of Medicine (IOM), accreditation councils and health professional bodies consider EBP as a core competency needed for health professionals [2–4]. Hence, EBP has become an integral part of undergraduate, postgraduate, and continuing health professional education curricula [5].

Despite the established interest in evidence-based practice (EBP) as a core competency for clinicians, evidence for how to effectively teach it remains suboptimal. Fifteen years ago, Hatala and Guyatt highlighted this: “the quantity and quality of the evidence for effectively teaching EBM are poor. Ironically, if one were to develop guidelines for how to teach EBM based on these results, they would be based on the lowest level of evidence” [6]. The disproportionate focus on critical appraisal compared to the other four steps in the EBP process (question formulation, searching, applying, and self-assessment) is a major shortcoming of the current literature for teaching EBP [6–8]. A review of 20 EBP educational interventions for undergraduate medical students found that these interventions stressed certain EBP steps (asking clinical question, acquire evidence, and critical appraisal) but pay less attention to others (apply, and assess and reflect) [9].

In addition, the lack of high-quality validated instruments to establish the effect of an educational intervention is also a shortcoming [6]. In 2006, Shaneyfelt et al. systematically identified 104 unique instruments for evaluating EBP teaching, the majority (90%) of which were not high quality instruments [10]. High quality instruments were those with established inter-rater reliability, objective outcome measures, and three or more types of established validity [10]. The ‘Fresno test of competence in evidence based medicine’ [11] and the Berlin Questionnaire [12] were the only high-quality instruments identified as evaluating EBP knowledge and skills across 3 of the 5 EBP steps [10]. In 2011, a classification rubric for EBP instruments in education (the CREATE framework) was developed to help EBP educators identify the best available EBP instruments for their educational needs [13].

Whether progress has been made to address these shortcomings (focus on EBP Step 3 and lack of high quality EBP instruments) is unclear. Therefore, we sought to systematically assess coverage of the five EBP steps in educational interventions, review the domains of outcomes measured in EBP educational interventions, and assess the psychometric properties of the instruments used in studies evaluating EBP educational interventions.

The review question was: “What are the contents of EBP educational interventions and how are the effect of EBP educational interventions measured?”

## Methods

We updated the search of a previously conducted systematic review of studies which evaluated the effect of EBP educational interventions (searched until March 2017) [14] to find additional studies and extract additional information on content, outcome domains and EBP instruments.

### Eligibility criteria

We included studies that were: controlled (studies with a separate control group, e.g. randomised controlled trials or non-randomised controlled trials); investigated the effect of EBP educational intervention which aimed to teach at least one component of the main EBP steps (of any format or mode - e.g. workshop, course, journal club); among health professionals (irrespective of the discipline or the level of training - undergraduate, postgraduate, or continuous professional education).

### Search strategy

We used a forward and backward citation analysis technique using the Web of Science database (until May 2017), with no language or publication year restrictions. Citation analysis can be used to identify all the articles that cited (“forward citation”) or were cited by (“backward citation”) the index articles. The index articles were the systematic reviews and primary studies included in an overview of systematic reviews of the effect of EBP teaching [15]. The Cochrane highly sensitive search filter for identifying controlled trials was applied [16]. In addition, the reference lists of included studies were also reviewed, and additional eligible studies were included for full-text assessment. Further, we searched the literature in Web of Science for published reliability and validity data of the EBP instruments reported in the included studies – using terms including the reference cited in the included article, the name of tool, and the authors involved in the development of the tool.

### Study selection

Titles and abstracts were screened to identify potentially eligible studies, and the full texts of these were assessed for inclusion by one of the authors (LA). Any concerns about study eligibility were discussed and resolved by all authors.

### Data extraction and analysis

We extracted data on study characteristics including publication year, country, sample size, design, and population. We extracted information on intervention content (EBP steps covered in the educational intervention) and categorised it into the five EBP steps [17]. We also extracted information on the outcome domains measured and organised them into the 7 categories according to Tilson et al. [13]: (i) Reaction to the EBP educational experience; (ii) Attitudes about EBP; (iii) Self-efficacy for conducting EBP; (iv) Knowledge about EBP principles; (v) Skills for performing EBP; (vi) Behaviour congruent with EBP as part of patient care; and (vii) Benefit to Patients associated with EBP. All three authors independently extracted data from a random sample of 20 articles and discussed extractions until consensus achieved. Data from the remaining articles were extracted by one of the authors (LA).

We also extracted information on the reliability and validity of the EBP instruments reported in the included studies – either from the included studies or retrieved articles from our search. The methods to evaluate the quality of instruments were based on those used by Shaneyfelt et al. [10] – high quality instruments should be supported by established interrater reliability (if applicable), objective (non–self-reported) outcome measures, and multiple (≥3) types of established validity evidence (including evidence of discriminative validity). Instruments that did not meet the criteria of high quality instruments were labelled low quality instruments. We considered the reliability and validity of an instrument as “established” if the corresponding statistical test was significant (e.g. quantitative assessment of the reliability and validity of an instrument was not enough).

## Results

**Fig. 1:**
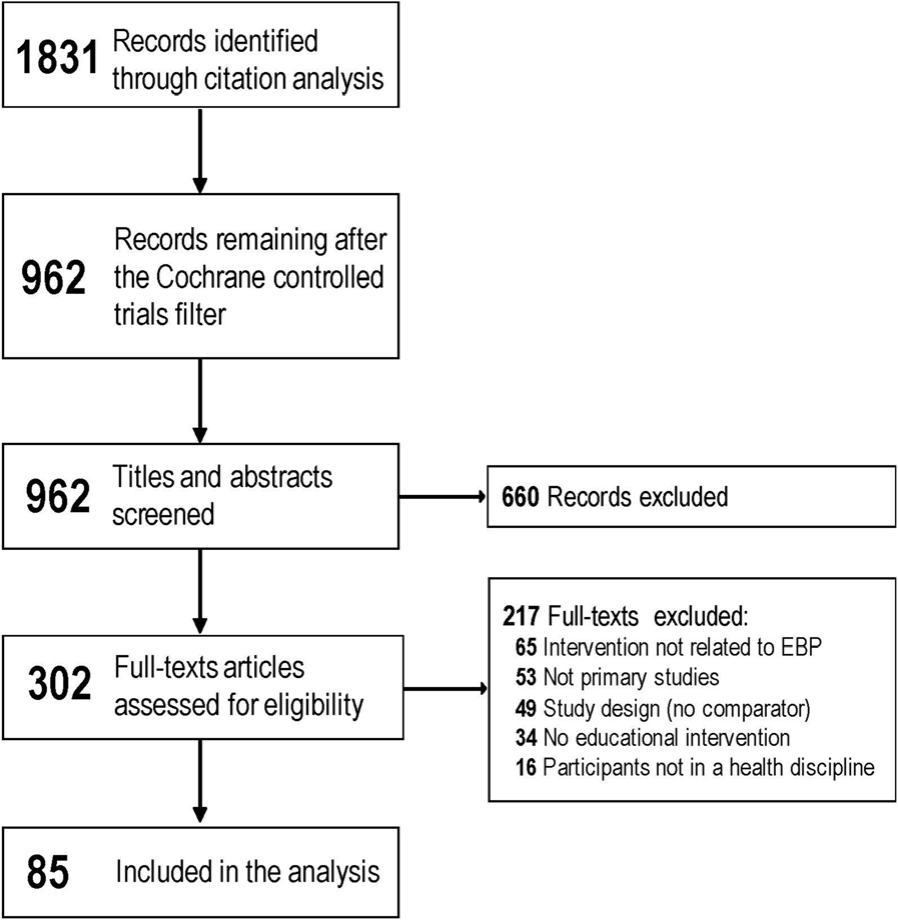
PRISMA flow chart of the systematic review

Of 1831 records retrieved by our search, 962 titles and abstracts were screened for eligibility. Of these, 302 full-text articles were screened for inclusion, and 217 articles were excluded (Fig. 1 shows the PRISMA flow chart). Of 85 included articles, 46 (54%) were randomized trials, 51 (60%) included postgraduate level participants, and 63 (75%) taught medical professionals. Table 1 shows characteristics of the included studies (See also Additional file 1 for a detailed description of each included study).

**Table 1:** Characteristics of the 85 included studies of EBP educational interventions

Characteristics	No. (%)
Location	
USA	35 (41%)
Europe	27 (32%)
Australia	7 (8%)
Canada	7 (8%)
Others	9 (11%)
Publication year	
< 2000	21 (25%)
2000–2004	18 (21%)
2005–2009	17 (20%)
≥ 2010	29 (34%)
Health disciplines	
Medical	63 (74%)
Nursing	8 (9%)
Allied health professions	14 (17%)
Training level	
Undergraduate	32 (38%)
Postgraduate	51 (60%)
Both	2 (2%)
Study design	
Randomised controlled trials	46 (54%)
Non-randomised controlled trials	39 (46%)

### Content coverage of EBP steps in included studies

**Fig. 2:**
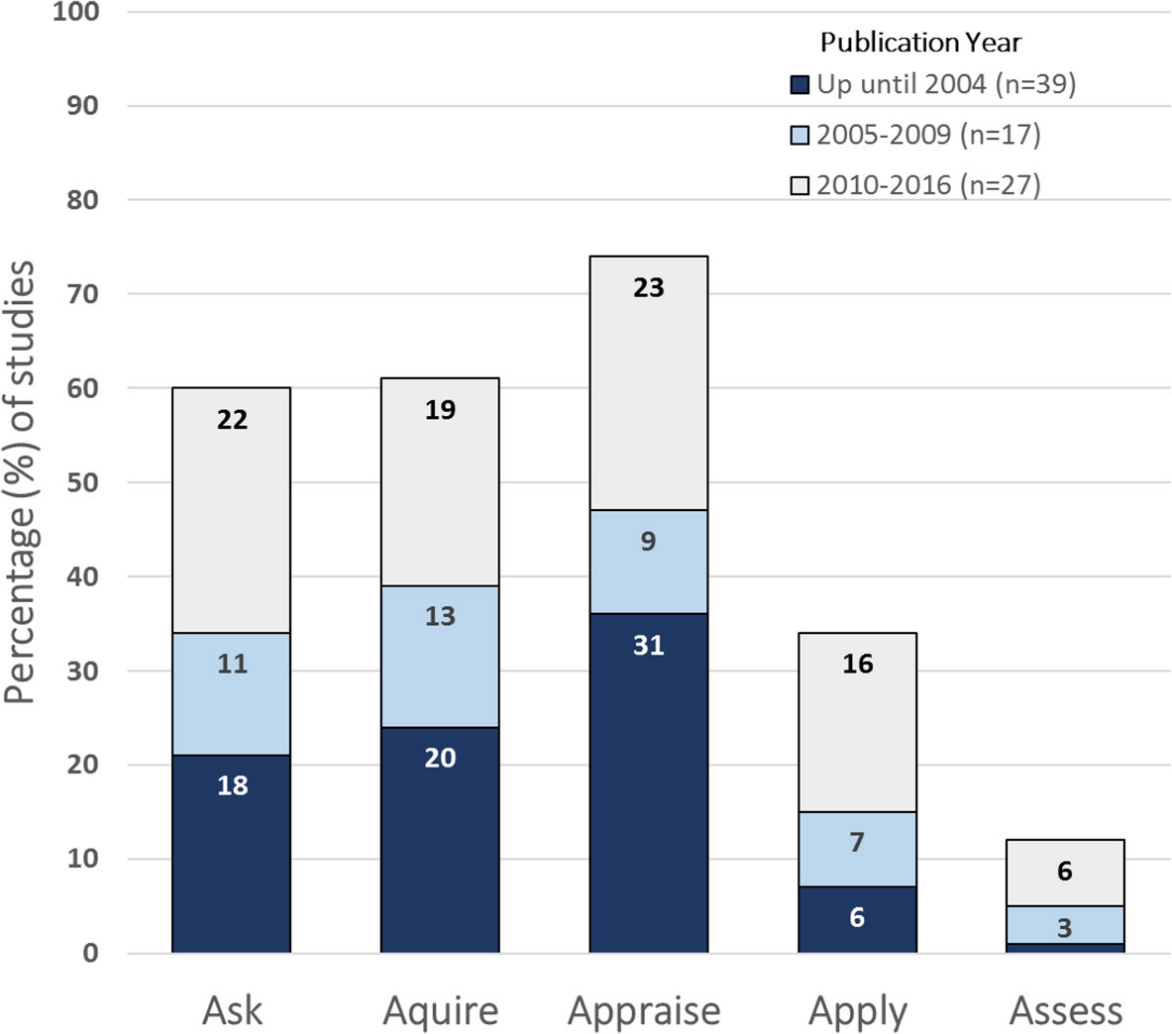
Percentage (numbers in bars) of studies which teach each of the 5 EBP steps (1: ask; 2: acquire; 3: appraise; 4: apply; 5: assess), grouped by publication year

EBP step 3 (critical appraisal of evidence) was the step taught most frequently in EBP educational interventions (*n* = 63; 74%), followed by step 2 (acquiring the evidence; *n* = 52; 63%) and step 1 (asking a clinical question; *n* = 51; 61%) (Fig. 2). About one-third of the studies (*n* = 30; 36%) covered only one of the five EBP steps, most commonly step 3 (critical appraisal of evidence). Only 10 (12%) studies covered all five EBP steps. However, the proportion of studies which taught all five steps increased over time - from 1 study (of 39; 3%) in years before 2004 to 6 studies (of 27; 22%) in 2010–2016, with a particular increase in coverage of steps 4 and 5.

### Outcome domains measured and quality of EBP instruments

Of the 85 included studies, 52 (61%) evaluated EBP skills, 39 (46%) knowledge, 35 (41%) attitudes, 19 (22%) behaviours, 15 (18%) self-efficacy, and 7 (8%) measured students’ reaction to the educational experience. None measured benefits to patients associated with EBP.

High-quality instruments (achieved ≥3 types of established validity evidence) were used across: 14 (29%) of 52 studies that measured EBP skills; 14 (41%) out of 39 studies that measured EBP knowledge; and 8 (26%) out of 35 studies that measured EBP attitude. None of the instruments used to measure EBP self-efficacy and behaviour were of high quality. Table 2 shows the overall outcome domains measured and quality of EBP instruments used in the included studies.

**Table 2:** Outcome domains and psychometric properties of instruments used in studies of EBP educational interventions (*n* = 85)

	Reaction to EBP Teaching Delivery	Attitude	Self-efficacy	Knowledge	Skills	Behaviors	Patient Benefit
Of 85 included studies, number measuring this outcome domain	7	35	15	39	52	19	0
Studies using previously developed instruments	0/7 (0)	24/35 (69)	5/15 (33)	24/39 (62)	20/52 (38)	7/19 (37)	0/0 (0)
Participant self-reported measure	7/7 (100)	35/35 (100)	15/15 (100)	0/39 (0)	0/52 (0)	18/19 (95)	0/0 (0)
Published/reported psychometric properties							
Inter-rater reliability^a^	0/7 (0)	0/35 (0)	0/15 (0)	8/39 (21)	15/52 (38)	2/19 (11)	0/0 (0)
Content validity^a^	0/7 (0)	12/35 (34)	2/15 (13)	19/39 (49)	15/52 (38)	2/19 (11)	0/0 (0)
Internal validity^a^	0/7 (0)	20/35 (57)	5/15 (33)	26/39 (67)	17/52 (44)	8/19 (42)	0/0 (0)
Responsive validity^a^	0/7 (0)	8/35 (23)	1/15 (7)	11/39 (28)	10/52 (26)	1/19 (5)	0/0 (0)
Discriminative validity^a^	0/7 (0)	9/35 (26)	4/15 (27)	15/39 (38)	16/52 (41)	0/19 (0)	0/0 (0)
Criterion validity^a^	0/7 (0)	4/35 (11)	1/15 (7)	2/39 (5)	1/52 (3)	2/19 (11)	0/0 (0)
Instrument ≥3 types of established validity^a^	0/7 (0)	8/35 (23)	0/15 (0)	14/39 (36)	14/52 (27)	0/19 (0)	0/0 (0)

^a^considered ‘established’ and counted if the corresponding statistical test was significant. *Abbreviation*: *EBP* Evidence-based practice

Definitions*: inter-rater reliability*, the degree to which the measurement is free from measurement error; *content validity*, external review of the instrument by EBP experts; internal validity, includes both internal consistency (i.e. the degree of the interrelatedness among the items) and dimensionality (i.e. factor analysis to determine if the instrument measured a unified latent construct); *responsive validity*, ability to detect the impact of EBP; *discriminative validity,* ability to discriminate between participants with different levels of EBP; *criterion validity*, the relationship between the instrument scores and participants’ scores on another instrument with established psychometric properties

Presented as number (%) of included studies within each measured outcome domain

### High-quality instruments used in EBP educational studies

Of the 24 previously developed instruments that were used across all included studies, 6 (25%) instruments were rated as high quality (Table 3). Four of these (Fresno Test [11], Berlin Questionnaire [12], Taylor et al. [18], and Assessing Competency in EBP “ACE” tool [19]) were used to measure both EBP knowledge and skills. The other two were used to measure either EBP knowledge [20] or skills [21]. The Fresno Test, Berlin Questionnaire, and Assessing Competency in EBP “ACE” tool evaluated three of the five EBP steps (ask, acquire, and appraise and interpret). Taylor et al. evaluated EBP step 2 and 3 (acquire, and appraise and interpret), Utrecht questionnaire evaluated EBP step 3 and 4 (appraise and interpret, apply) [20], and MacRae et al. evaluated EBP step 3 only [21]. Table 3 summarises high-quality instruments used in EBP educational interventions.

**Table 3:** High quality instruments (achieved ≥3 types of established validity evidence) used in some of the included studies

Source instrument name and date	Instrument development	Outcome domain	EBP steps*	Instrument Description	Type of validity/reliability evidence
Ramos et al. 2003 [11] (Fresno Test)	43 Family practice residents and faculty members, 53 experts in EBM, and 19 family practice teachers (US).	Knowledge and skills	1,2,3	The Fresno test was originally developed and validated to assess medical professionals’ knowledge and skills in EBP, however, it has been adapted for use in other health disciplines (e.g. occupational therapy [37], physical therapy [38], and pharmacy [39]) and in other languages (e.g. Brazilian-Portuguese version [40]). It consists of two clinical scenarios with 12 open-ended questions. It needs about 40–60 min to complete and 10–15 min to mark using standardised grading rubrics (scores ranged from 0 to 21).	Content Internal consistency Discriminative Inter-rater reliability
Fritsche et al. 2002 [12]; Akl et al. 2004 [41] (Berlin Questionnaire)	43 experts in EBM, 20 medical students, 203 participants in EBP course (Germany); 49 Internal medicine residents in Non-randomized controlled trial of EBP curriculum (US)	Knowledge and skills	1, 2, 3	The Berlin questionnaire was developed and validated to assess EBP knowledge and skills in medicine, but has been translated and validated in other languages (e.g. Dutch [42]). It consists of two separate sets of 15 multiple choice questions with 5 response option each, which mainly focus on epidemiological knowledge and skills (scores ranged from 0 to 15).	Content Internal consistency Discriminative Responsive
Ilic et al. 2014 [19] (ACE tool)	342 medical students: 98 EBM-novice, 108 EBM-intermediate and 136 EBM advanced (Australia).	Knowledge and Skill	1,2,3	ACE tool was also developed and validated to assess EBP knowledge and skills in medicine and consists of 15 dichotomous-choice (yes or no) questions, based on a short patient scenario, a relevant search strategy and a hypothetical article extract (Scores ranged from 0 to 15).	Content Internal consistency Discriminative Responsive Inter-rater reliability
Taylor et al. 2001 [18]; Bradley et al. 2005 [43]; Sánchez-Mendiola et al. 2012 [44] (Spanish version)	152 health care professionals (UK); 175 medical students (Norway); 289 medical students (Mexico)	Attitude, knowledge, skill	2,3	Part I: 6 multiple-choice questions each with three items, with 3 potential answers, each requiring a true, false, or “don’t know” response; the range of scores is − 18 to 18. Part II: 7 statements related to the use of evidence in practice, and each scored using a five-point Likert scale; the range of scores is 7 to 35.	Content Internal consistency Discriminative Responsive
Kortekaas et al. 2017 [20] (Utrecht questionnaire “U-CEP”) in Dutch	219 general practice (GP) trainees, 20 hospital trainees, 20 GP supervisors, and 8 expert academic GPs or clinical epidemiologists (The Netherlands)	Knowledge	3,4	Two formats: two sets of 25 comparable questions (6 open-ended and 19 multiple-choice questions) and a combined set of 50 questions. Multiple-choice question scored 1 for correct and 0 for incorrect answer. Open-ended questions scored 0 to 3. Scores ranged from 0 to 33 for set A and 0–34 for set B.	Content Internal consistency Discriminative Responsive Inter-rater reliability
MacRae et al. 2004 [21]	44 Surgery residents (Canada)	Skill	3	3 Journal articles, each followed by a series of short-answer questions and 7-point scales to rate the quality of elements of the study design; short-answer questions based on cards from an EBP textbook (Evidence-Based Medicine: How To Practice And Teach It [1])	Internal consistency Discriminative Responsive

* EBP steps (1: ask; 2: acquire; 3: appraise; 4: apply; 5: assess)

## Discussion

Our systematic review of controlled studies of EBP educational interventions found that only 12% of interventions taught content that covered all five EBP steps. Over half of the 85 EBP educational studies did not use a high quality instrument to measure their outcomes of interest. Only six high quality EBP instruments were used in the included studies, but none were designed to evaluate all five EBP steps.

Although few of interventions taught content that covered all five EBP steps, increasing recognition of the importance of the “apply” step of EBP through processes such as shared decision making may account for increased coverage of the fourth step in more recent years [22].

This is the first systematic review that we are aware of to evaluate the instruments used in EBP educational studies. However, there are a number of previous systematic reviews that have identified and evaluated all available EBP instruments (whether used in controlled educational studies or not), and these also found only a small number of high quality instruments. Shaneyfelt et al. identified 104 unique instruments for evaluating the effectiveness of EBP training, the majority of which were developed or tested with medical students or trainees. Seven of the 104 instruments identified in Shaneyfelt and colleagues’ review were recognised as high quality instruments (i.e. supported by established inter-rater reliability, objective outcome measures, and three or more types of established validity) [10].

Thomas et al. found that only the Fresno test has been assessed with more than one group of family physician residents and reported a full set of validity and reliability measures [23]. Leung et al. identified 24 different instruments for measuring EBP knowledge, skills and attitude among nurses, and found that only one (the revised EBPQ [24]) had adequate validity for measuring knowledge, skills and attitudes in EBP [25]. Oude et al. found that of 160 EBP instruments for assessing EBP behaviour (i.e. only one of the seven outcome domains that we addressed) among health professionals, no instruments have established validity and reliability that assessed all five EBP steps [26].

The CREATE framework proposed guidance for developing new EBP instruments by purposively classifying the assessment domains (e.g. self-efficacy, knowledge, skills) and types (e.g. self-report, performance assessment) within the five EBP steps [13]. Development and agreement on a core set of valid and reliable recommended instruments to measure outcome domains is essential to reliably establish the effectiveness of EBP educational interventions. This would include evaluation of previously developed validated EBP instruments (e.g. Fresno test, Berlin Questionnaire) across health disciplines, and translation of these tools into other languages using standardised methods. EBP instruments measuring the clinicians’ use of EBP processes in practice (e.g. frequency of search for evidence) are needed. Innovative new approaches to evaluate EBP teaching (e.g. objective structured clinical examination [27], use of standardised patients within the context of a performance-based examination [28], use of audio-recording in clinics [29]) that balance robustness with feasibility should be explored. Despite the ultimate goal of EBP education being to improve the quality of care and patient outcomes, it is nearly impossible to measure this [30]. In a systematic review of 599 research articles published in three major medical education journals, patient outcomes accounted in only 0.7% of all articles [31]. Some of the factors that can impede measuring the impact of EBP education on the quality of care and patient outcomes include: the impact of educational interventions is often latent and distant; and the dominant role of the overarching team and health care system on quality of care and patient outcomes [32, 33].

Similar to previous studies [7, 8], we found that the majority of evaluated EBP educational interventions focus on critically appraising evidence (EBP Step 3), often to the exclusion of other steps (i.e. apply and reflect). If EBP educational interventions remain mostly focused on teaching how to locate and appraise evidence, research evidence may be poorly translated into clinical practice. Instead, greater emphasis should be placed on teaching learners how to apply and the evidence in collaboration with individual patients such as through shared decision making. An International consensus statement of core competencies in EBP for health professionals has been recently developed and includes 68 core competencies that should be taught in EBP educational programs [34]. This may help to harmonise the content of EBP educational interventions, and with possibly flow-on effect to the measured outcomes.

This systematic review has a number of limitations. We may have missed some relevant studies by using citation analysis as the searching method. However, the accuracy rate of citation analysis has been found to be acceptable [35, 36]. For instance, using this technique, Janssens and Gwinn identified 94% [75–100%] of all articles included in 10 systematic reviews that were originally used the conventional search strategy [35]. Therefore, overall conclusions are unlikely to be affected. Screening and data extraction were performed by one author, and multiple researchers independently extracted data from only a random sample of 20 articles. Another limitation is that we might have inaccurately rated the psychometrics properties of EBP instruments as for some instruments this judgement was limited by inadequate reporting of the results of psychometric testing.

Our findings have a number of implications for health educators and researchers. EBP educators should identify specific assessment tools (for formative and summative use) that provide accurate, reliable, and timely evaluation of the EBP education being provided and map these assessment tools to the EBP domains targeted. If necessary, educators may need to develop appropriate assessment tools designed specifically to evaluate the identified gaps in EBP assessment tools (e.g. EBP step 4: apply), and recognise the need to evaluate the psychometric properties of any tools developed.

## Conclusions

After over two decades of EBP teaching which has spread across professions and clinical settings, the majority of evaluated EBP educational interventions remain focussed on critically appraising evidence (EBP Step 3), often to the exclusion of other steps (i.e. apply and reflect). There are few validated instruments that have been developed and utilised in EBP educational intervention studies; and these predominantly focus on certain domains (i.e. knowledge and skills) and EBP steps (i.e. appraise). This might limit the ability to evaluate the impact of EBP educational interventions.

## Additional file

Additional file 1: Characteristics and detailed descriptions of included studies. (DOC 55 kb)

## Abbreviations

ACE: Assessing Competency in EBP
CREATE: Classification Rubric for EBP Assessment Tools in Education
EBP: Evidence-Based Practice
EBPQ: Evidence-based Practice Questionnaire
IOM: Institute Of Medicine
PRISMA: Preferred Reporting Items for Systematic Reviews and Meta-Analyses
